# The role of GpsB in *Staphylococcus aureus* cell morphogenesis

**DOI:** 10.1128/mbio.03235-23

**Published:** 2024-02-06

**Authors:** Sara F. Costa, Bruno M. Saraiva, Helena Veiga, Leonor B. Marques, Simon Schäper, Marta Sporniak, Daniel E. Vega, Ana M. Jorge, Andreia M. Duarte, António D. Brito, Andreia C. Tavares, Patricia Reed, Mariana G. Pinho

**Affiliations:** 1 Instituto de Tecnologia Química e Biológica António Xavier, Universidade NOVA de Lisboa, Oeiras, Portugal; Carnegie Mellon University, Pittsburgh, Pennsylvania, USA

**Keywords:** *Staphylococcus aureus*, elongasome, GpsB, morphogenesis

## Abstract

For decades, cells of the Gram-positive bacterial pathogen *Staphylococcus aureus* were thought to lack a dedicated elongation machinery. However, *S. aureus* cells were recently shown to elongate before division, in a process that requires a shape elongation division and sporulation (SEDS)/penicillin-binding protein (PBP) pair for peptidoglycan synthesis, consisting of the glycosyltransferase RodA and the transpeptidase PBP3. In ovococci and rod-shaped bacteria, the elongation machinery, or elongasome, is composed of various proteins besides a dedicated SEDS/PBP pair. To identify proteins required for *S. aureus* elongation, we screened the Nebraska Transposon Mutant Library, which contains transposon mutants in virtually all non-essential staphylococcal genes, for mutants with modified cell shape. We confirmed the roles of RodA/PBP3 in *S. aureus* elongation and identified GpsB, SsaA, and RodZ as additional proteins involved in this process. The *gpsB* mutant showed the strongest phenotype, mediated by the partial delocalization from the division septum of PBP2 and PBP4, two penicillin-binding proteins that synthesize and cross-link peptidoglycan. Increased levels of these PBPs at the cell periphery versus the septum result in higher levels of peptidoglycan insertion/crosslinking throughout the entire cell, possibly overriding the RodA/PBP3-mediated peptidoglycan synthesis at the outer edge of the septum and/or increasing stiffness of the peripheral wall, impairing elongation. Consequently, in the absence of GpsB, *S. aureus* cells become more spherical. We propose that GpsB has a role in the spatio-temporal regulation of PBP2 and PBP4 at the septum versus cell periphery, contributing to the maintenance of the correct cell morphology in *S. aureus*.

**IMPORTANCE:**

*Staphylococcus aureus* is a Gram-positive clinical pathogen, which is currently the second cause of death by antibiotic-resistant infections worldwide. For decades, *S. aureus* cells were thought to be spherical and lack the ability to undergo elongation. However, super-resolution microscopy techniques allowed us to observe the minor morphological changes that occur during the cell cycle of this pathogen, including cell elongation. *S. aureus* elongation is not required for normal growth in laboratory conditions. However, it seems to be essential in the context of some infections, such as osteomyelitis, during which *S. aureus* cells apparently elongate to invade small channels in the bones. In this work, we uncovered new determinants required for *S. aureus* cell elongation. In particular, we show that GpsB has an important role in the spatio-temporal regulation of PBP2 and PBP4, two proteins involved in peptidoglycan synthesis, contributing to the maintenance of the correct cell morphology in *S. aureus*.

## INTRODUCTION

*Staphylococcus aureus* is a Gram-positive bacterial pathogen, responsible for a high number of antibiotic-resistant infections, and is currently the second major cause of death by antibiotic-resistant infections (1). For decades, *S. aureus* cells were thought to lack a dedicated elongation machinery, and *S. aureus* was often mentioned as a canonical example of truly spherical cocci. Rods, which have a cylindrical shape, undergo cell elongation before the synthesis of a septum that divides the mother cell in two identical daughter cells. In these organisms, cell elongation and division are organized by two cytoskeletal proteins, the bacterial actin homolog MreB and the tubulin homolog FtsZ, respectively, which coordinate the assembly of two multiprotein complexes, the elongasome or Rod complex and the divisome (2–4). These complexes include cell wall synthesis proteins and organize synthesis of new peptidoglycan at the lateral wall and at the division septum.

*S. aureus* cells lack an MreB homolog and were previously thought to lack a canonical elongasome (5). The view that *S. aureus* had only one cell wall synthesis machinery that made peptidoglycan at the division septum was present in work by G. Satta in the 1990s (6). This work proposed a “two-competing-sites model” for peptidoglycan assembly which predicted that there were two types of cocci: one that synthesized peptidoglycan only at the septum (which would include *S. aureus*) and another that also synthesized peptidoglycan for lateral wall elongation (which would include ovococci such as *Enterococcus faecium* or *Streptococcus agalactiae*) (6). In these studies, *S. aureus* was unable to elongate under various conditions that promoted elongation of ovococci, such as the presence of antibiotics that inhibit septation (6). However, the recent development of super-resolution microscopy techniques allowed imaging of the small (~1 µm in diameter) staphylococcal cells, with sufficient resolution to observe minor morphological changes that occur during the cell cycle. This showed that *S. aureus* cells do undergo slight elongation, which is reflected in an increase in the ratio of the longer cell axis (perpendicular to the division septum) to the shorter cell axis (overlapping the septum) during the cell cycle (7). The *S. aureus* cell cycle can be divided in three different phases: phase 1, before initiation of division septum synthesis; phase 2, during which septum synthesis occurs; and phase 3, during which cells have a complete septum, undergoing maturation, before splitting in two identical daughter cells (7). Cell volume increases during the entire cell cycle, while cell elongation occurs mostly during phases 1 and 3 (7). We have recently shown that this elongation process is dependent on the presence of the shape elongation division and sporulation (SEDS)/penicillin-binding protein (PBP) pair RodA/PBP3, proteins with glycosyltransferase activity responsible for glycan strand synthesis and transpeptidase activity responsible for peptidoglycan crosslinking, respectively (8). Together, these proteins catalyze peptidoglycan synthesis, including at the sidewall of the septal region, resulting in slight cell elongation (8). This midcell synthesis is reminiscent to that observed in ovococcoid bacteria such as *Streptococcus pneumoniae*, which also lack an MreB homolog (9, 10), but different from elongation of rod-shaped bacteria such as *Escherichia coli* or *Bacillus subtilis* where MreB polymerizes into short filaments that move processively in a circumferential direction, perpendicular to the long axis of the rod, organizing the elongasome machinery and leading to peptidoglycan incorporation over the entire length of the cell (11–13). Besides having a dedicated SEDS/PBP pair, rod elongasome also includes two transmembrane proteins, MreC and MreD, proposed to couple intracellular MreB to extracellular PBPs (14) and RodZ, which interacts with other elongasome components (15) and may modulate MreB filament density (16).

Given the relative complexity of elongasomes in rods, it seemed unlikely that elongation in *S. aureus* would require just RodA and PBP3, the only two proteins so far identified as having a role in this process. We hypothesized that *S. aureus* elongation was not essential during growth in rich medium, as deletion of both *rodA* and *pbp3* (previously called *pbpC*) genes was not lethal in these conditions (8). Therefore, to identify additional factors required for *S. aureus* elongation, we screened by microscopy the Nebraska Transposon Mutant Library (NTML), composed of mutants with a *bursa aurealis* transposon insertion in virtually every non-essential gene of the methicillin-resistant *S. aureus* (MRSA) strain JE2 (17), for mutants with cells with decreased eccentricity.

## MATERIALS AND METHODS

### Bacterial growth conditions

*S. aureus* strains were grown at 37°C in tryptic soy broth (TSB, Difco) with agitation or on plates of tryptic soy agar (Difco). When necessary, culture media were supplemented with appropriate antibiotics chloramphenicol 10 µg/mL, erythromycin 10 µg/mL (Sigma-Aldrich, during intermediate step in the construction of deletion mutants) or erythromycin 25 µg/mL (NTML mutants), kanamycin 150 µg/mL (Apollo Scientific, overnight cultures) or 50 µg/mL (cultures for microscopy), or a combination of kanamycin (50 µg/mL) with neomycin (50 µg/mL, Apollo Scientific), with 100 µg/mL 5- bromo-4-chloro-3-indolyl-β-D-galactopyranoside (X-Gal, VWR), or with 0.1 µM CdCl_2_ (Sigma-Aldrich). *E. coli* strains were grown at 37°C in Luria-Bertani broth (LB, Difco) or on LB agar (Difco) supplemented with ampicillin 100 µg/mL (Sigma-Aldrich) when required.

### Microscopy screening of the NTML

Batches of 48 mutants of the NTML (17) were imaged, with parental strain JE2 imaged before and after the first and last groups of eight mutants, respectively. Each strain was grown overnight at 37°C in TSB supplemented with erythromycin 25 µg/mL, except for JE2 which was grown without antibiotic. Each overnight culture was back diluted 1:200 in TSB and grown to an OD_600_ of 0.8. From each culture, a 300 µL aliquot was incubated with 0.4 µL of both Nile red (10 mg/mL, Invitrogen) and Hoechst 33342 (5 mg/mL, Invitrogen) for 5 min at 37°C with shaking. Samples were pelleted and resuspended in 10 µL of a solution of 1:3 (vol/vol) TSB/phosphate-buffered saline (PBS, NaCl 137 mM, KCl 2.7 mM, Na_2_HPO_4_ 10 mM, KH_2_PO_4_ 1.8 mM). One microliter of each sample was mounted on a layer of 1.2% (wt/vol) agarose in 1:3 (vol/vol) TSB/PBS placed on a glass plate (Bio-Rad Mini-PROTEAN Short Plate) with a coverslip placed on top of each sample. Eight samples were imaged per glass plate, using a Zeiss Axio Observer microscope equipped with a Plan-Apochromat 100×/1.4 oil Ph3 objective, a Retiga R1 CCD camera (QImaging), a white-light source HXP 120 V (Zeiss), and Metamorph 7.5 software (Molecular Devices). The filters (Semrock) Brightline TXRED-4040B (Nile red) and Brightline DAPI-1160A (Hoechst 33342) were used for image acquisition. Phase contrast and widefield fluorescence microscopy images (minimum of five fields of view per mutant) were acquired with an exposure time of 100 ms for all channels.

### Analysis of cell morphology of NTML mutants

The eHooke software (18) was used to automatically perform image segmentation of cells from each mutant (at least five fields of view per mutant) and to measure morphological parameters of individual cells, namely, area, perimeter, axis sizes, eccentricity, and irregularity. This was followed by a visual inspection of the images by at least two users to qualitatively evaluate the quality of segmentation results. Data from images that were not correctly segmented were not considered for further analysis. Mutants were ranked by eccentricity values (which can theoretically vary between 0 and 1), calculated according to equation 1, where “a” and “b” are the semi-major and semi-minor axes, respectively, defined as the major and minor axes of the smallest rectangle that can contain each cell (18).


(equation 1) $$Eccentricity=\sqrt{1-\frac{b^{2}}{a^{2}}}$$

### Construction of plasmids and bacterial strains

The plasmids and bacterial strains used in this study are described in Tables S1 and S2, respectively. The primers used are listed in Table S3.

Deletion mutants were constructed using the thermosensitive plasmid pMAD (19). The upstream and downstream regions of the genes SAUSA300_1090, SAUSA300_2249 (*ssaA*), SAUSA300_0128, SAUSA300_1337 (*gpsB*), SAUSA300_1113 (*pknB*), SAUSA300_1175 (*rodZ*), and SAUSA300_0629 (*pbp4*) were amplified from *S. aureus* genomic DNA using the primers identified in Table S3. Subsequently, these fragments were cloned into pMAD plasmid in the SmaI site, in *E. coli* DC10B, using NEBuilder HiFi DNA assembly mix (New England BioLabs) through the Gibson assembly technique (20) creating pMAD-Δ*1090*, pMAD-Δ*ssaA*, pMAD-Δ*0128*, and pMAD-Δ*pknB* (see Table S1). For pMAD-Δ*gpsB*, pMAD-Δ*rodZ*, and pMAD-PBP4-KO, the amplified upstream and downstream regions were joined by overlap PCR and cloned into pMAD using the restriction sites NcoI/BamHI or EcoRI/NcoI. Plasmids pMAD-Δ*pbpC* (8)*,* pMAD-Δ*rodA* (8), pΔ*mreD* (21), and pΔ*mreC* (21) were already available.

The plasmids were extracted using a miniprep kit (NZYTech); the constructs were confirmed by Sanger sequencing (STAB VIDA) and then electroporated into *S. aureus* RN4220 at 30°C using erythromycin and X-gal selection (22). Blue colonies were selected to produce a phage lysate using phage 80α (23), and the plasmids were transduced to *S. aureus* JE2 or COL. Constructs were integrated into the chromosome at 43°C, as previously described (19). Cells were then incubated at 30°C, and colonies where the plasmid had undergone excision were selected and tested by PCR, resulting in strains JE2 Δ*1090*, JE2 Δ*ssaA*, JE2 Δ*pbpC*, JE2 Δ*rodA*, JE2 Δ*0128*, JE2 Δ*mreD*, JE2 Δ*gpsB*, JE2 Δ*pknB*, JE2 Δ*mreC*, JE2 Δ*rodZ*, JE2 Δ*pbp4*, and COL Δ*pbp4*. Using the same procedure, pMAD-Δ*gpsB* vector was transduced into strains COL, COL Δ*pbp4,* ColsGFP-PBP1 (24), BCBPM073 (25), ColsGFP-PBP3 (24), COLpPBP4-YFP (26), and COL EzrA-sGFP (27) to delete *gpsB*, resulting, respectively, in strains COL Δ*gpsB*, COL Δ*pbp4*Δ*gpsB*, ColsGFP-PBP1 Δ*gpsB*, ColsGFP-PBP2 Δ*gpsB*, ColsGFP-PBP3 Δ*gpsB*, COLpPBP4-YFP Δ*gpsB*, and COL EzrA-sGFP Δ*gpsB*. Deletion of each gene was confirmed by PCR using primers in Table S3 followed by Sanger sequencing, across the deleted region.

To complement the deletion mutants with plasmid-encoded copies of the corresponding genes, each gene was amplified by PCR, using primers indicated in Table S3, and cloned into the pCNX (7) or pCN51 (28) plasmid under the control of P*_cad_* promoter, using restriction enzymes SmaI and EcoRI for *mreC* and *mreD* fragments and NEBuilder HiFi DNA assembly mix (New England BioLabs), in the SmaI restriction site, through the Gibson assembly technique (20) for the remaining fragments. The following plasmids were constructed: pCNX*1090*, pCNX*ssaA*, pCNX*0128*, pCNX*mreD*, pCNX*gpsB*, pCN51*gpsB,* pCNX*pknB*, pCNX*mreC*, pCNX*rodZ*, and pBCBPM115 (pCNX encoding *pbp4*) (see Table S1). After confirmation of the insert sequence in pCNX or pCN51 by PCR and Sanger sequencing, DNA of plasmids with the correct insert was extracted and electroporated into *S. aureus* RN4220 at 37°C using kanamycin and erythromycin selection, respectively (22). Each plasmid was then transduced into the corresponding deletion mutant using phage 80α, generating JE2 Δ*1090* pCNX*1090*, JE2 Δ*ssaA* pCNX*ssaA*, JE2 Δ*0128* pCNX*0128*, JE2 Δ*mreD* pCNX*mreD*, JE2 Δ*gpsB* pCNX*gpsB*, JE2 Δ*pknB* pCNX*pknB*, JE2 Δ*mreC* pCNX*mreC*, JE2 Δ*rodZ* pCNX*rodZ,* JE2 Δ*pbp4* pCNX*pbp4*, and COL Δ*gpsB* pCNX*gpsB*. As a control, the pCNX empty vector was transduced into *S. aureus* JE2 strain, generating JE2 pCNX. Strains ColsGFP-PBP2 Δ*gpsB* pCNX*gpsB* and ColsGFP-PBP2 pCNX*gpsB* were obtained by transducing plasmid pCNX*gpsB* into ColsGFP-PBP2 Δ*gpsB* and ColsGFP-PBP2, respectively. Strains COLpPBP4-YFP Δ*gpsB* pCN51*gpsB* and COLpPBP4-YFP pCN51*gpsB* were obtained by transducing plasmid pCN51*gpsB* into COLpPBP4-YFP Δ*gpsB* and COLpPBP4-YFP, respectively.

### Analysis of *S. aureus* growth

The growth of COL, COL Δ*gpsB*, JE2, and JE2 Δ*gpsB* strains was analyzed in a 96-well plate reader (Biotek Synergy Neo2). Overnight cultures were diluted 1:1,000 in fresh media, 200 µL of each culture were added to wells of a 96-well plate, and growth was followed at 37°C with agitation for 18 ½ hours. The OD_600_ was measured every 30 min.

### Fluorescence microscopy and cell morphology image analysis

For imaging, *S. aureus* strains were grown overnight at 37°C in TSB with selective antibiotics, diluted to an OD_600_ of 0.05, and grown at 37°C with selective antibiotics and appropriate inducers until cultures reached an OD_600_ of 0.5–0.6. When required, cells were labeled for 5 min at 37°C, with agitation, with DNA dye Hoechst 33342 (1 μg/mL, Invitrogen) and membrane dye Nile Red (5 µg/mL, Invitrogen). The cells were centrifuged for 1 min at 10,000 rpm in a benchtop centrifuge (Eppendorf 5430) and resuspended in PBS, and 1 µL of this cell suspension was placed onto a gel pad composed of 1.2% agarose (TopVision, Thermo Fisher Scientific) prepared in PBS and mounted on a microscopy slide.

Images were acquired with a Zeiss Axio Observer Z1 microscope equipped with a Plan-Apochromat 100×/1.40 oil Ph3 objective with numerical aperture 0.55, HXP 120 V Illuminator (Zeiss), and Photometrics CoolSNAP HQ camera (Roper Scientific, Inc.) and controlled by a ZEN software (Zeiss). The filters Brightline TXRED-4040B and Brightline DAPI-1160A (Semrock) were used for image acquisition of cells labeled with Nile red and Hoechst 33342, respectively. Phase-contrast images and widefield microscopy images were acquired with 100 ms exposure time. At least 10 images per mutant were acquired, and three biological replicates were performed for each mutant.

Automated analysis of microscopy images was performed using eHooke (18) to measure morphological parameters, including cell eccentricity, and to automatically assign the cell cycle phase to each analyzed cell. Eccentricity was calculated using Equation 1 described above. Data were plotted using GraphPad Prism 8 (GraphPad Software).

### Assessment of FtsZ dynamics

To measure FtsZ treadmilling speed, COL EzrA-sGFP and COL EzrA-sGFP Δ*gpsB* strains were grown overnight, in triplicate, in TSB and diluted 1:200 in fresh TSB followed by incubation with shaking at 37°C. Exponentially growing cells (OD_600_ of 0.6–0.8) were harvested by centrifugation for 1 min at 9,300 × *g*, resuspended in 30 µL fresh TSB, and spotted on a pad of 1.5% molecular biology grade agarose (Bio-Rad) in M9 minimal medium (KH_2_PO_4_ 3.4 g/L, VWR; K_2_HPO_4_ 2.9 g/L, VWR; di-ammonium citrate 0.7 g/L, Sigma-Aldrich; sodium acetate 0.26 g/L, Merck; glucose 1% [wt/vol], Merck; MgSO_4_ 0.7 mg/L, Sigma-Aldrich; CaCl_2_ 7 mg/L, Sigma-Aldrich; casamino acids 1% [wt/vol], Difco; MEM amino acids 1×, Thermo Fisher Scientific; MEM vitamins 1×, Thermo Fisher Scientific) mounted in a Gene Frame (Thermo Fisher Scientific) on a microscope slide. Imaging was performed in a DeltaVision OMX SR microscope equipped with a hardware-based focus stability (HW UltimateFocus) and an environmental control module set to 37°C. Z-stacks of three images with a step size of 500 nm were acquired every 3 seconds for 3 min using a 488-nm laser (100 mW, at 10% maximal power) with an exposure time of 50 ms. Maximum intensity projection (MIP) of three images from each Z-stack and subsequent image deconvolution were performed for each time frame in the software SoftWoRx. All 61 time frames were aligned using NanoJ-Core drift correction (29) and then used to perform MIP for the drawing of 1-pixel freehand lines over EzrA-sGFP signal in images of newborn sister cells that are still attached via the septum of the mother cell, in which nascent Z-rings appear sparse and Dshaped. Space-time kymographs were generated by extracting fluorescence intensities from individual time frames along drawn freehand lines using the software Fiji (30, 31). FtsZ treadmilling speed was calculated (in nanometers per second) by determining the slope of diagonals spanning the entire width of generated kymographs.

### Analysis of the localization of PBPs and of peptidoglycan synthesis

To localize fluorescent derivatives of PBPs 1–4, strains ColsGFP-PBP1, BCBPM073 (encoding sGFP-PBP2), ColsGFP-PBP3, and COLpPBP4-YFP, the corresponding *gpsB* deletion mutants ColsGFP-PBP1 Δ*gpsB*, ColsGFP-PBP2 Δ*gpsB*, ColsGFP-PBP3 Δ*gpsB*, COLpPBP4-YFP Δ*gpsB*, complemented strains ColsGFP-PBP2 Δ*gpsB* pCNX*gpsB* and COLpPBP4-YFP Δ*gpsB* pCN51*gpsB*, and strains overexpressing *gpsB* ColsGFP-PBP2 pCNX*gpsB* and COLpPBP4-YFP pCN51*gpsB* were grown in TSB 37°C (plus 0.1 µM CdCl_2_ for strains containing pCNX*gpsB* and pCN51*gpsB*) to OD_600_ of 0.5. Images were acquired with the Zeiss Axio Observer Z1 microscope described above, with filters Brightline GFP-3035D and Brightline YFP-2427A (Semrock) to image GFP and YFP derivatives, respectively, with 4,000- to 5,000-ms exposure time.

To evaluate localization of peptidoglycan synthesis activity, *S. aureus* cells of parental strains COL/JE2, *gpsB* mutants COL/JE2 Δ*gpsB*, and complemented strains COL/JE2 Δ*gpsB* pCNX*gpsB* were grown in TSB (plus 0.1 µM CdCl_2_ for the complemented strains) at 37°C, in triplicate, to an OD_600_ of 0.4 and labeled with fluorescent 7-hydroxycoumarin-3-carboxylic acid-amino-D-alanine (HADA) (32) at 0.1 mM for 30 min at 37°C, with agitation. Cells were then washed with PBS and placed on an 1.5% agarose pad mounted on a microscopy slide. Images were acquired with the Zeiss Axio Observer Z1 microscope described above using the filter Brightline DAPI-1160A (Semrock), with 100 ms exposure time.

For image analysis, phase 3 cells (with complete septum) were selected, and the ratio of the fluorescence signal at the septum (considering only the 25% brightest pixels) versus the cell periphery was calculated using eHooke (18). Statistical analysis was performed using a two-sided Mann-Whitney *U* test, performed using the Python package SciPy version 1.10.1 (33).

### Bacterial two-hybrid (BTH) assays

Plasmids pUT18-GpsB, pKNT25-GpsB, and pKT25-GpsB were constructed by amplifying by PCR the *gpsB* gene, using primers described in Table S3, and cloning this insert into the BTH plasmids pUT18C, pKNT25, and pKT25. The correct sequence of the insert was confirmed by Sanger sequencing.

Plasmids pUT18 and pKT25 (negative control); p18Zip and p25Zip (positive control); various combinations of constructed plasmids pUT18-GpsB, pKNT25-GpsB, and pKT25-GpsB and plasmids p18PBP2 and p25PBP2 (34) were co-transformed into the reporter strain BTH101 (35). Cells were plated on LB agar supplemented with 40 µg/mL X-Gal, 0.5 mM IPTG, 100 µg/mL ampicillin, and 50 µg/mL kanamycin and incubated for 43 h at 30°C.

Individual colonies from each transformation with a pair of plasmids were resuspended in 10 µL of LB, and 3 µL was spotted on MacConkey agar containing 1% maltose, 0.5 mM IPTG, 100 µg/mL ampicillin, and 50 µg/mL kanamycin and incubated for 18 h at 30°C. Three independent experiments were conducted.

## RESULTS

### Screening of NTML for mutants with reduced eccentricity

*S. aureus* cells undergo minor elongation during the cell cycle (7, 8). To the best of our knowledge, only two proteins, PBP3 and RodA (8), have been identified so far as being required for the elongation process. To identify new determinants required for *S. aureus* elongation, we labeled the 1920 NTML transposon insertion mutants in non-essential genes of JE2 (17), as well as the parental strain JE2, with a membrane dye and a DNA dye and imaged them by fluorescence microscopy. At least five fields of view were imaged for each strain, resulting in a library of over 10,000 images. Morphological parameters of each cell were automatically determined using eHooke software (18), and mutants were ranked from lowest (more spherical) to highest (more elongated) average cell eccentricity.

The top seven mutants with lowest eccentricity were selected for further studies: *SAUSA300_1090*, *lgt*, *ssaA*, *pbpC* (aka *pbp3*), *rodA*, *SAUSA300_0128*, and *mreD* (Table S4; Fig. S1). The fact that the third and fourth positions in the ranking of increasing eccentricity were occupied by the genes encoding for RodA and PBP3, required for elongation (8), validates the screening. Repeating the imaging of selected transposon mutants did not confirm the decreased eccentricity of the *lgt* mutant, which was, therefore, considered a false positive from the screening and not further studied.

From the top 100 hits of the eccentricity screening, we selected two other candidates, GpsB and PknB, which we reasoned could have a role in elongation based on the function of these proteins in other organisms. GpsB contributes to the control of elongation/division in *B. subtilis* by shuttling between the septum and the lateral wall, together with penicillin-binding protein PBP1 (36). In *S. pneumoniae*, depletion of GpsB results in the formation of elongated, enlarged cells, presumably because GpsB mediates septal ring closure (37, 38). Given that the *B. subtilis* serine/threonine kinase PrkC phosphorylates GpsB (39), we also included in our study the main serine/threonine protein kinase present in *S. aureus*, PknB.

Two additional mutants lacking proteins with reported roles in elongation of other bacterial species, MreC and RodZ, were also included in this study. MreC, together with MreD, has a role in cell elongation through the coordination of peripheral peptidoglycan synthesis in *B. subtilis* and *S. pneumoniae* (14, 40). A previous study did not identify morphology changes in *S. aureus* mutants lacking MreD and MreC, but their eccentricity was not determined (21). Given that the *mreD* mutant was a top hit in the screening, we decided to include also the *mreC* mutant in this study, although it ranked lower in the screening (Table S4). RodZ is a non-essential component of the elongasome (also known as Rod complex) of *E. coli* or *B. subtilis*, and *rodZ* deletion results in cell shape changes from rod to round or ovoid (41–43). Although a *rodZ* transposon mutant is absent from the NTML, we were able to generate a *rodZ* deletion mutant in JE2, showing that it is not an essential gene in this *S. aureus* strain.

Finally, we also included the mutant lacking PBP4 because we previously showed that this protein is involved in peripheral peptidoglycan synthesis in *S. aureus* (7). We reasoned that PBP4 could have a role in cell elongation via insertion of peripheral peptidoglycan, despite its low ranking in the screening for cell eccentricity (Table S4).

**Fig 1:**
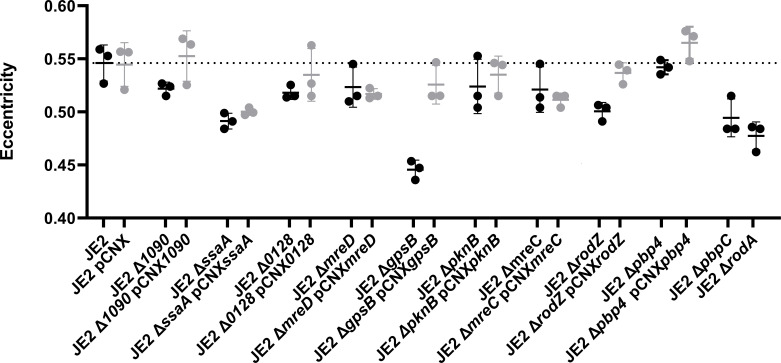
*S. aureus* mutants with reduced eccentricity. Cell eccentricity was measured in cells with a complete septum (cell cycle phase 3, *n* = 550 cells/replicate, three replicates per strain) of selected mutants (black circles). Each mutant was complemented with the corresponding gene encoded in pCNX replicative plasmid (gray circles). The empty vector was introduced in the parental strain JE2. Black and gray lines represent the mean eccentricity and standard deviation of the three replicates of each strain. The average eccentricity of JE2 cells is indicated by the dashed line.

To confirm the results from the screening and test if the morphology changes in the transposon mutants were not due to polar effects on downstream genes, new mutants were made by individually deleting each selected gene mentioned above from the genome of strain JE2. Furthermore, each deletion mutant was complemented with a plasmid expressing the corresponding gene. Deletion mutants and corresponding complemented strains were labeled with membrane and DNA dyes and imaged by widefield fluorescence microscopy. The eccentricity was measured only in cells with a closed septum (cell cycle phase 3 cells), since elongation is more pronounced in this phase (7, 8) (Fig. 1). As expected, cells of all tested mutants, except for *pbp4*, had lower eccentricity (cells were more spherical) than cells of the parental strains JE2. However, for mutants in *ssaA*, *SAUSA300_0128*, *mreC*, and *mreD*, this phenotype was not fully complemented by the corresponding plasmid-encoded gene, possibly due to the lack of native gene regulation or protein overexpression. The strongest phenotype, which was readily complemented by plasmid-encoded gene, was observed for cells of the *gpsB* deletion mutant, which was, therefore, selected for further studies. Whole genome sequencing of the *gpsB* deletion mutant confirmed the deletion of the gene and identified only one single-nucleotide polymorphism (SNP) leading to a synonymous mutation (G51>T) in the *gatB* gene (SAUSA300_1880) encoding aspartyl/glutamyl-tRNA amidotransferase subunit B. We confirmed that deletion of *gpsB* does not impair growth and that the role of GpsB in cell elongation was not unique to JE2, as it was observed in the background of another well-studied MRSA strain, COL (Fig. S2).

### Lack of GpsB does not alter FtsZ treadmilling speed

**Fig 2:**
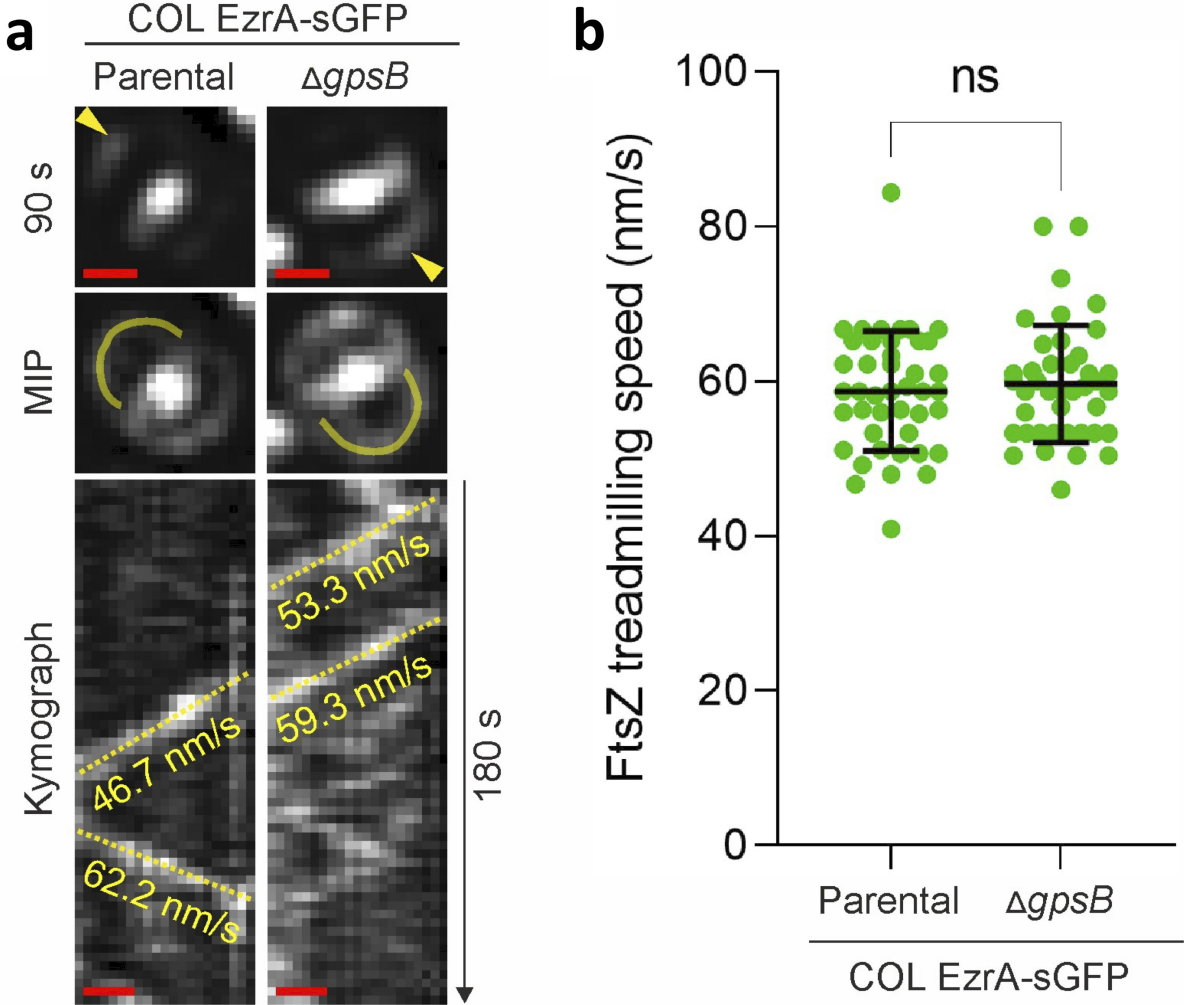
GpsB does not affect FtsZ treadmilling speed *in vivo*. (**a**) Representative epifluorescence images of EzrA-sGFP in pre-divisional cells with nascent Z-rings of strains COL EzrA-sGFP (parental) and COL EzrA-sGFP Δ*gpsB* at a selected time point and throughout a 180-second time series (MIP, maximum intensity projection). Representative cells with nascent Z-rings are shown for each strain. Yellow arrow heads indicate an EzrA-sGFP patch whose change in localization was followed over time. Kymographs were generated by extracting fluorescence intensity values along indicated yellow lines. Yellow dashed lines in kymographs indicate the slopes used to calculate EzrA-sGFP movement speed (in nanometers per second), a proxy for FtsZ treadmilling. Scale bars, 0.5 µm. (**b**) FtsZ treadmilling speed measured in indicated strains. Data are represented as scatter plots in which the middle line represents the mean, and the top and bottom lines show the standard deviation of slopes determined from diagonal lines in kymographs. Experiments were performed in triplicate. Statistical analysis was performed using a two-tailed Mann-Whitney *U* test (*P* = 0.5179); COL EzrA-sGFP, *n*_total_ = 130. COL EzrA-sGFP Δ*gpsB*, *n*_total_ = 132; ns, non-significant.

GpsB is a small coiled-coil protein, which localizes at midcell, previously reported to be essential in *S. aureus* strain SH1000 (44). However, a transposon mutant in *gpsB* is present in the NTML (17) and in additional reported transposon screenings (45), and deletion mutants of *gpsB* have been recently reported (46) including in SH1000 (47). We could also easily delete *gpsB* from both COL and JE2 strains, indicating that *gpsB* is dispensable for growth in various *S. aureus* strains. The function of GpsB in *S. aureus* is still not fully elucidated, but it has been proposed to promote stabilization of the Z-ring at the onset of cell division (although in a mutant that required GpsB for growth [44]). This was suggested to result in higher local concentration of FtsZ, activation of its GTPase activity, and triggering of FtsZ treadmilling (44). In *S. aureus,* FtsZ treadmilling is essential during the early stages of cytokinesis (24). We hypothesized that regulation of FtsZ treadmilling activity by GpsB could modulate peptidoglycan synthesis at the septum, causing the morphology changes observed in the *gpsB* mutant. To measure FtsZ treadmilling speed, we used as a proxy a functional fusion of superfast GFP (sGFP) (48) to EzrA, a direct interaction partner of FtsZ. We have previously shown that FtsZ and EzrA undergo similar movement dynamics, sensitive to FtsZ inhibitor PC190723 (24). Our quantitative analysis focused on the early stages of cytokinesis, in which nascent Z-rings appear sparse and Dshaped, in newborn sister cells that are still attached via the septum of the mother cell (Fig. 2a), given that GpsB was proposed to stabilize FtsZ at the onset of cell division (44). We observed that in TSB-rich medium at 37°C, FtsZ filaments/bundles moved at the same speed in the parental strain COL EzrA-sGFP (57.7 ± 7.8 nm/s; *n* = 130) and the *gpsB* deletion mutant COL EzrA-sGFP Δ*gpsB* (58.7 ± 8.9 nm/s; *n* = 132) (Fig. 2b), indicating that GpsB does not affect FtsZ treadmilling. Therefore, *S. aureus* cell morphology changes in the *gpsB* mutant are not mediated by regulation of FtsZ treadmilling.

### PBP2 and PBP4 partially delocalize from the septum in the absence of GpsB

**Fig 3:**
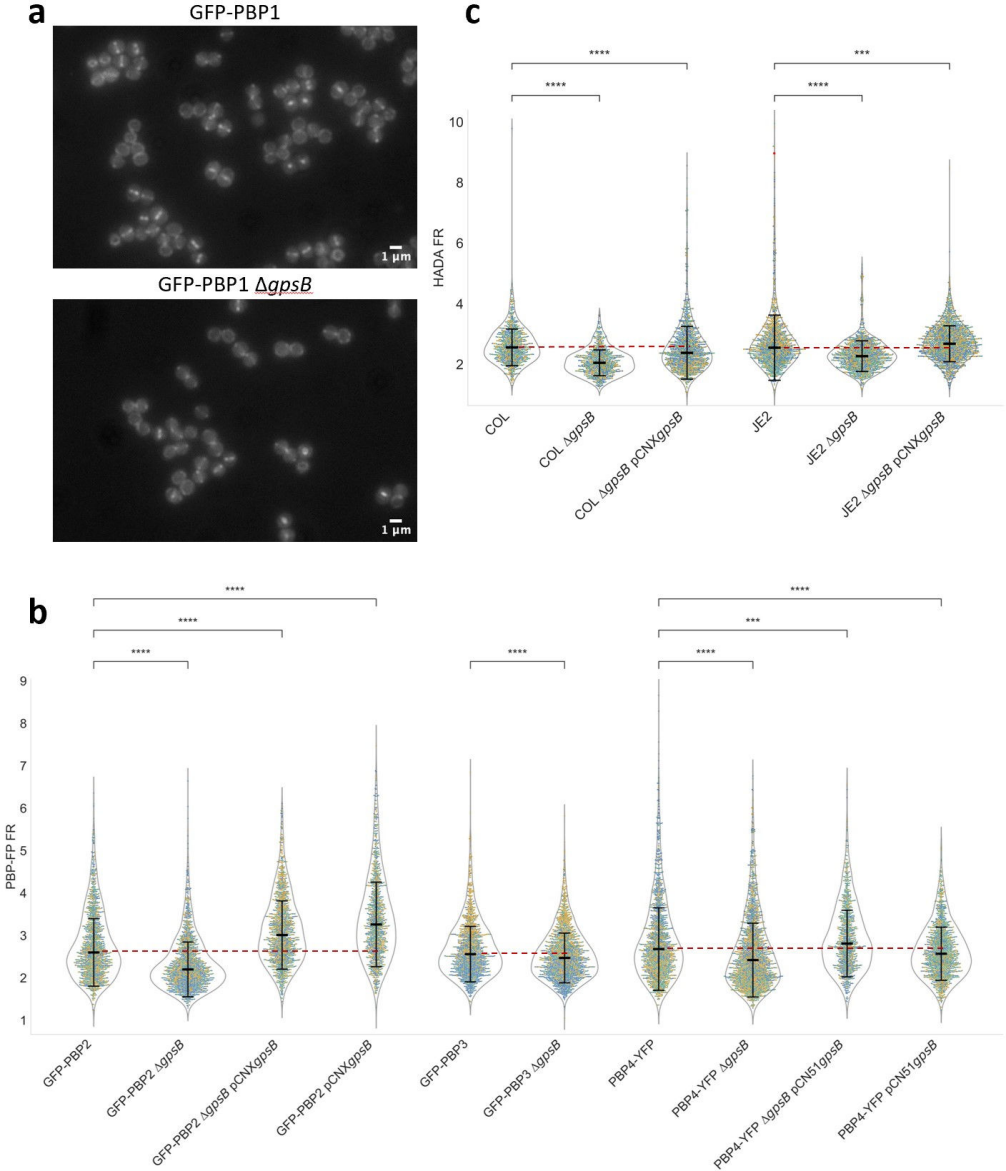
PBP2 and PBP4 partially delocalize from the septum in the absence of GpsB, leading to increased peptidoglycan synthesis at the cell periphery versus the septum. Strains ColsGFP-PBP1, BCBPM073 (encoding sGFP-PBP2), ColsGFP-PBP3, and COLpPBP4-YFP, expressing fluorescent derivatives of the four *S*. *aureus* native PBPs, the corresponding *gpsB* deletion mutants, the GpsB-complemented strains ColsGFP-PBP2 Δ*gpsB* pCNX*gpsB* and COLpPBP4-YFP Δ*gpsB* pCN51*gpsB,* and GpsB-overexpressing strains ColsGFP-PBP2 pCNX*gpsB* and COLpPBP4-YFP pCN51*gpsB* were imaged by fluorescence microscopy. (**a**) No change was observed in the localization pattern of the fluorescent derivatives of PBP1 in the absence of GpsB, with PBP1 localizing exclusively at the septum in ColsGFP-PBP1 and the corresponding *gpsB* deletion mutant. (**b**) PBP2 and PBP4 partially delocalized from the division septum in the absence of GpsB, while PBP3 showed very mild, if any, delocalization. PBP2 and PBP4 delocalization in the *gpsB* mutants is reverted upon complementation with plasmid encoded *gpsB*. PBP2 (but not PBP4) septal enrichment is further increased upon overexpression of GpsB. Graph shows the ratio of the signal of fluorescent derivatives of PBPs at the septum versus the peripheral membrane (PBP-FP FR), in cells with a complete septum. (**c**) Parental strains COL/JE2, *gpsB* mutants COL/JE2 Δ*gpsB*, and complemented strains COL/JE2 Δ*gpsB* pCNX*gpsB* cells were labeled for 30 min with HADA, a fluorescent derivative of D-alanine that is incorporated in peptidoglycan. Graph shows the ratio of the fluorescent signal of HADA at the septum versus the peripheral membrane (HADA FR), in cells with a complete septum. This ratio is lower for *gpsB* mutants indicating that there is higher peptidoglycan synthesis activity at the cell periphery versus the septum in the absence of GpsB, and reverts to levels close to those observed in the parental strains upon complementation with plasmid encoded *gpsB*. (**b and c**) Strains containing pCNX*gpsB* and pCN51*gpsB* were grown in the presence of 0.1 µM CdCl_2_ to induce *gpsB* expression. Experiments were done in triplicate with yellow, green, and blue dots corresponding to each replicate (n_total_ >1,150 for GFP-PBP2 samples, *n*_total_ > 1,300 for GFP-PBP3 samples, *n*_total_ > 975 for PBP4-YFP samples; in graph 3c, *n*_total_ >730 for COL strains and *n*_total_ > 1,065 for JE2 strains). Black lines show medians and standard deviations. Red dashed line indicates FR mean of parental strains to facilitate comparison with mutants. Statistical analysis was performed using a two-sided Mann-Whitney *U* test; ***0.0001 < *P* < 0.001, ****P < 0.0001.

Besides interacting with FtsZ (44), *S. aureus* GpsB also interacts with PBP4 through a signature GpsB recognition sequence, suggesting that it may link cell division and peptidoglycan synthesis (49, 50). If correct localization of one or more *S. aureus* PBPs was dependent on GpsB, then its absence could result in peptidoglycan incorporation at incorrect cellular locations, which could lead to morphological alterations. To test if this was the case, we deleted *gpsB* in four previously constructed strains expressing fluorescent fusions to each of the four *S*. *aureus* native PBPs, PBP1-4, and imaged the resulting strains by fluorescence microscopy. PBP1, an essential class b PBP with transpeptidase activity, was previously shown to localize at the septum. This localization was not altered in the absence of GpsB, as the sGFP-PBP1 fluorescence signal was present at the septum and absent from the peripheral membrane in both ColsGFP-PBP1 and ColsGFP-PBP1 Δ*gpsB* (Fig. 3a). For strains expressing fluorescent derivatives of PBP2, 3, and 4 (which are enriched at the septum but also present at the cell periphery), we calculated the ratio of the fluorescence signal at the septum versus the cell periphery (FR, Fig. 3b). PBP3 showed very mild, if any, delocalization, while FR for both PBP2 and PBP4 decreased in the absence of GpsB, indicating partial delocalization of these proteins from the septum to the cell periphery. PBP2 is the only bifunctional PBP in *S. aureus*, with both glycosyltransferase and transpeptidase activity, for synthesis of the glycan strands and crosslinking via peptide bridges, respectively (51). PBP4 is a low-molecular-weight PBP with transpeptidase activity, responsible for the high levels of crosslinking characteristic of *S. aureus*, shown to be involved in peptidoglycan synthesis at the cell periphery (7, 52). To confirm that PBP2 and PBP4 partial delocalization from the septum was in fact caused by lack of GpsB, we complemented strains ColsGFP-PBP2 Δ*gpsB* and COLpPBP4-YFP Δ*gpsB* with plasmid-encoded *gpsB*, and in both cases, septal enrichment was recovered (Fig. 3b). PBP2 septal enrichment was further increased upon overexpression of GpsB, although we could not observe the same for PBP4 (Fig. 3b). However, the importance of PBP4 peripheral activity for the decreased eccentricity of cells lacking GpsB is clear, as deleting both *pbp4* and *gpsB* results in cells with eccentricity similar to those of the parental strain COL (Fig. S3).

**Fig 4:**
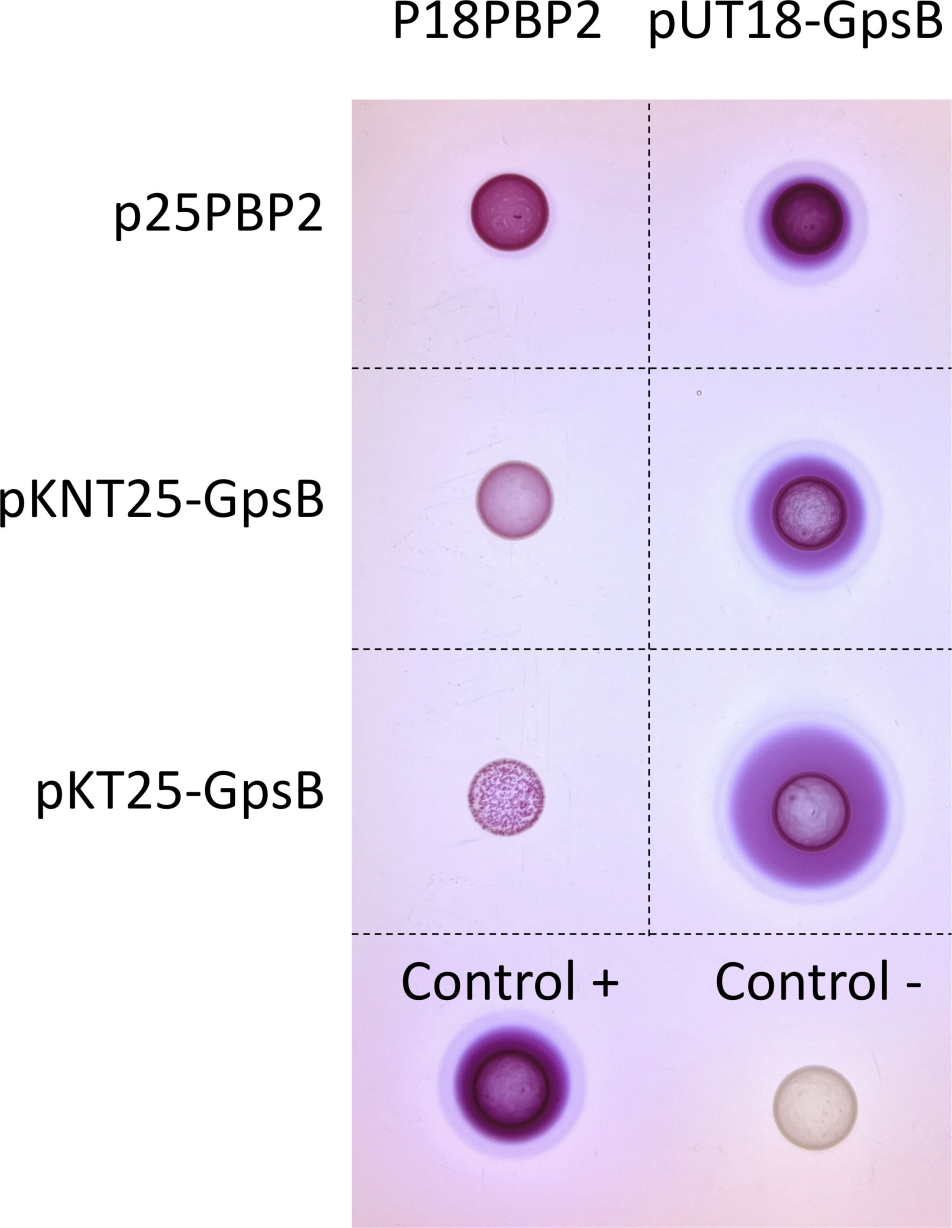
GpsB and PBP2 interact in a bacterial two-hybrid assay. A bacterial two-hybrid assay detected self-interaction of PBP2 and GpsB, as well as an interaction between PBP2 and GpsB. Plasmids p18Zip and p25Zip were used as a positive control, and empty vectors pUT18C and pKT25 were used as negative control. *E. coli* BTH101 strains containing pairs of plasmids indicated in the figure were spotted in maltose-containing MacConkey where red color indicates an interaction.

As mentioned above, PBP4 has been shown to interact with GpsB (49). However, to the best of our knowledge, this has not been shown for PBP2. We, therefore, tested, by bacterial two-hybrid assay, if we could detect an interaction between PBP2 and GpsB (Fig. 4). Our data confirm that GpsB and PBP2 self-interact, as previously shown (49, 53), and suggest that GpsB interacts with PBP2, although this protein lacks a GpsB recognition motif (49). Therefore, the role of GpsB in the regulation of PBP2 and PBP4 localization may be mediated by protein-protein interactions.

Partial delocalization of PBP2 and PBP4 to the cell periphery should lead to increased peptidoglycan synthesis over the entire cell surface. We labeled strains COL and JE2 as well as the corresponding COL/JE2 Δ*gpsB* mutants and COL/JE2 Δ*gpsB* pCNX*gpsB* complemented strains, with HADA, a fluorescent derivative of D-alanine which is specifically incorporated into the pentapeptide chain of peptidoglycan (32). We observed that the ratio between peptidoglycan synthesis at the septum versus the cell periphery decreases in the absence of GpsB, a phenotype that is reversed in the complemented strains (Fig. 3c). This increased peripheral synthesis may increase the stiffness of the peripheral wall and/or override the RodA/PBP3-mediated synthesis at the outer edge of the septum, previously shown to be required for *S. aureus* elongation (8), resulting in rounder cells in the *gpsB* mutants.

## DISCUSSION

Elongation of *S. aureus* is dispensable *in vitro*, as mutants lacking the RodA/PBP3 pair of peptidoglycan synthases, required for elongation, exhibit no growth defects in laboratory conditions such as TSB-rich medium and 37°C (8). However, the retention of an (albeit minor) elongation capacity throughout evolution implies potential advantages in alternative conditions. Interestingly, in a murine model of osteomyelitis, an infection primarily caused by *S. aureus*, staphylococcal cells were observed as submicron rod-shaped bacteria within the canaliculi of live cortical bone (54). This suggests that deformation/elongation of *S. aureus* may be required for migration in bones during osteomyelitis (54). Furthermore, while wild-type USA300 *S. aureus* cells were capable of propagation through a nanopore narrower than the diameter of the cells, mutants lacking PBP3 or PBP4 showed reduced propagation through the nanopores, suggesting impaired ability to undergo the required deformation (55, 56). These mutants also failed to invade and colonize the canaliculi of cortical bone (55, 56), indicating that the ability to deform/elongate is required during osteomyelitis. Therefore, understanding the mechanism of elongation of *S. aureus* may be relevant not only for expanding our knowledge of cocci morphogenesis, but also to gain a better understanding of how this bacterial pathogen causes an infection that is often regarded as incurable (54).

In this work, we analyzed the impact of individually deleting 11 non-essential genes on the eccentricity of *S. aureus* cells. Two of these genes encoded RodA and PBP3, the SEDS/PBP pair previously reported to be involved in *S. aureus* elongation (8). The remaining nine mutants all had decreased eccentricity, indicating that elongation of *S. aureus* may be a complex process requiring multiple determinants. However, the phenotype of five of these mutants was very mild: two mutants lacking proteins of unknown function (SAUSA300_1090 and SAUSA300_0128) and three mutants lacking MreC or MreD, two proteins with a well-studied role in elongation of rods (14, 57), and the serine/threonine protein kinase PknB (Fig. 1). In agreement with its low ranking in the initial NLTM screening for altered eccentricity, the mutant lacking PBP4, a peptidoglycan transpeptidase involved in peripheral peptidoglycan synthesis in *S. aureus* (7), showed similar eccentricity to that of the parental strain JE2 (Fig. 1). The remaining three mutants showed a stronger phenotype, with eccentricity being reduced by close to 10%. These include mutants lacking (i) RodZ, a member of the elongasome of rods in which it interacts with MreC, MreD, and RodA/bPBPs, possibly modulating MreB filament density in *B. subtilis* (16) and linking MreB filaments to the elongation peptidoglycan synthases (15). RodZ is also present in ovococcoid bacteria such as *S. pneumoniae*, where it seems to act as a scaffold protein of the elongasome despite the absence of MreB (58), a role that may be similar to its role in *S. aureus*; (ii) SsaA, an autolysin with amidase activity (59). For peptidoglycan to expand in surface area, a process presumably required for cell elongation, both synthesis and hydrolysis are required to allow for insertion of new material and relaxation of old material. SsaA may have a role in the relaxation of the peptidoglycan mesh at midcell during elongation, but further studies are required to test this hypothesis; (iii) GpsB, a small membrane associated protein, is widely conserved in firmicutes and proposed to coordinate peptidoglycan synthesis for cell growth and division by binding cytoplasmic mini-domains of PBPs to ensure their correct subcellular localization (50). In *S. pneumoniae*, which lacks MreB, similarly to *S. aureus*, GpsB is part of a molecular switch that orchestrates peripheral and septal peptidoglycan synthesis, and cells elongate in the absence of GpsB (37, 38). The *S. aureus* mutant lacking GpsB was the only one in this study with cells more spherical (lower eccentricity) than those of the RodA/PBP3 mutants, pointing to an important role of this protein in staphylococcal morphogenesis. We, therefore, focused on understanding the mechanism by which GpsB regulates cell shape in *S. aureus*. Interestingly, data concurrent with ours show that *gpsB* deletion in *S. aureus* SH1000 also results in more spherical cells (47).

GpsB was previously suggested to regulate FtsZ treadmilling in *S. aureus* (44), which could alter peptidoglycan synthesis activity at the septum. However, we did not see any change in FtsZ treadmilling speed upon deletion of *gpsB*. Alternatively, given that GpsB was recently shown to interact with PBP4 in *S. aureus* (49), we hypothesized that GpsB could modulate *S. aureus* cell shape by directing PBPs to the cell periphery or to the septum. In agreement with this hypothesis, we observed that both PBP2 (which we showed also interacts with GpsB) and PBP4 partially delocalize from the septum to the peripheral wall in the absence of GpsB. This results in higher peptidoglycan synthesis activity in the cell periphery, which was confirmed by increased incorporation of the fluorescent D-amino acid HADA in the peptidoglycan of the peripheral wall versus the septum. Localized peptidoglycan synthesis at the sidewall of the septal region is required for cell elongation in *S. aureus* (8). We propose that the increased peptidoglycan synthesis at the cell periphery in the absence of GpsB overrides the localized RodA/PBP3-mediated peptidoglycan synthesis at midcell, leading to more spherical cells. Conversely, in wild-type cells that express GpsB, PBP2 and PBP4 activity is more restricted to the septum, and expansion of the peripheral wall occurs mostly near the outer edge of the septum, allowing mild elongation of *S. aureus* cells. It is also possible that increased peripheral activity of PBP2 and PBP4 in a *gpsB* mutant and the consequent increase in peptidoglycan crosslinking lead to a stiffer peripheral wall that does not expand to elongate, resulting in rounder and smaller cells in the absence GpsB.

Our data do not necessarily imply that GpsB is a member of an elongasome complex but that it has a role in ensuring correct morphogenesis of *S. aureus* by contributing to the spatio-temporal regulation of PBPs.

## Data Availability

Data will be made available upon reasonable request to the corresponding author.

## SUPPLEMENTAL MATERIAL

Supplemental material mbio.03235-23-s0001.docx Supplemental tables and figures.

ASM does not own the copyrights to Supplemental Material that may be linked to, or accessed through, an article. The authors have granted ASM a non-exclusive, world-wide license to publish the Supplemental Material files. Please contact the corresponding author directly for reuse.
