# Supplementary material for: The role of GpsB in Staphylococcus aureus cell morphogenesis
Source: mBio. 2024 Feb 6;15(3):e03235-23. doi: 10.1128/mbio.03235-23 (PMC10936418; doi:10.1128/mbio.03235-23)
Supplement: Supplemental material — Supplemental tables and figures. [file mbio.03235-23-s0001.docx]

Supplementary Information

The role of GpsB in *Staphylococcus aureus* cell morphogenesis

Sara F. Costa^1,#^, Bruno M. Saraiva^1,#^, Helena Veiga^1,#^, Leonor B. Marques^1,#^, Simon Schäper^1^, Marta Sporniak^1^, Daniel E. Vega^1^, Ana M. Jorge^1^, Andreia M. Duarte^1^, António D. Brito^1^, Andreia C. Tavares^1^, Patricia Reed^1^, Mariana G. Pinho^1,*^

^1^Instituto de Tecnologia Química e Biológica António Xavier, Universidade NOVA de Lisboa, Oeiras, Portugal.

# These authors have contributed equally to this work

Table 1: Plasmids used in this work

| Plasmid | Description | Reference |
| --- | --- | --- |
| pMAD | *E. coli* /*S. aureus* shuttle vector with a thermosensitive origin of replication for Gram-positive bacteria, Amp^R^, Ery^R^, *lacZ* | (1) |
| pMAD-Δ*1090* | pMAD with upstream and downstream regions of SAUSA300_1090 to construct a null mutant, Amp^R^, Ery^R^, *lacZ* | This work |
| pMAD*-*Δ*ssaA* | pMAD with upstream and downstream regions of SAUSA300_2249 (*ssaA*) to construct a null mutant, Amp^R^, Ery^R^, *lacZ* | This work |
| pMAD*-*Δ*pbpC* | pMAD with upstream and downstream regions of SAUSA300_1512 (*pbpC,* encoding PBP3) to construct a null mutant, Amp^R^, Ery^R^, *lacZ* | (2) |
| pMAD-Δ*rodA* | pMAD with upstream and downstream regions of SAUSA300_2040 (*rodA*) to construct a null mutant, Amp^R^, Ery^R^, *lacZ* | (2) |
| pMAD-Δ*0128* | pMAD with upstream and downstream regions of SAUSA300_0128 to construct a null mutant, Amp^R^, Ery^R^, *lacZ* | This work |
| pΔ*mreD* | pMAD with upstream and downstream regions of SAUSA300_1604 (*mreD*) to construct a null mutant, Amp^R^, Ery^R^, *lacZ* | (3) |
| pMAD-Δ*gpsB* | pMAD with upstream and downstream regions of SAUSA300_1337 (*gpsB*) to construct a null mutant, Amp^R^, Ery^R^, *lacZ* | This work |
| pMAD-Δ*pknB* | pMAD with upstream and downstream regions of SAUSA300_1113 (*pknB*) to construct a null mutant, Amp^R^, Ery^R^, *lacZ* | This work |
| pΔ*mreC* | pMAD with upstream and downstream regions of SAUSA300_1605 (*mreC*) to construct a null mutant, Amp^R^, Ery^R^, *lacZ* | (3) |
| pMAD-Δ*rodZ* | pMAD with upstream and downstream regions of SAUSA300_1175 (*rodZ*) to construct a null mutant, Amp^R^, Ery^R^, *lacZ* | This work |
| pMAD-PBP4-KO | pMAD with upstream and downstream regions of SAUSA300_0629 (*pbpD* encoding PBP4) to construct a null mutant, Amp^R^, Ery^R^, *lacZ* | This work |
| pCNX | *E. coli* /*S. aureus* shuttle vector containing a cadmium inducible P_cad_ promoter; Amp^R^, Kan^R^ | (4) |
| pCN51 | *E. coli* /*S. aureus* shuttle vector containing a cadmium inducible P_cad_ promoter; Amp^R^, Ery^R^ | (5) |
| pCNX*1090* | pCNX encoding SAUSA300_1090 under the control of P_cad_ | This work |
| pCNX*ssaA* | pCNX encoding SAUSA300_2249 (*ssaA*) under the control of P_cad_ | This work |
| pCNX*0128* | pCNX encoding SAUSA300_0128 under the control of P_cad_ | This work |
| pCNX*mreD* | pCNX encoding SAUSA300_1604 (*mreD*) under the control of P_cad_ | This work |
| pCNX*gpsB* | pCNX encoding SAUSA300_1337 (*gpsB*) under the control of P_cad_ | This work |
| pCNX*pknB* | pCNX encoding SAUSA300_1113 (*pknB*) under the control of P_cad_ | This work |
| pCNX*mreC* | pCNX encoding SAUSA300_1605 (*mreC*) under the control of P_cad_ | This work |
| pCNX*rodZ* | pCNX encoding SAUSA300_1175 (*rodZ*) under the control of P_cad_ | This work |
| pBCBPM115 | pCNX encoding SAUSA300_0629 (*pbpD* encoding PBP4) under the control of P_cad_ | (4) |
| pCN51*gpsB* | pCN51 encoding SAUSA300_1337 (*gpsB*) under the control of P_cad_ | This work |
| pUT18 | BTH plasmid; C-term *cyaA*T18 fusions; Amp^r^ | (6) |
| pUT18C | BTH plasmid; N-term *cyaA*T18 fusions; Amp^r^ | (6) |
| pKT25 | BTH plasmid; N-term *cyaA*T25 fusions; Kan^r^ | (6) |
| pKNT25 | BTH plasmid; C-term *cyaA*T25 fusions; Kan^r^ | (6) |
| p18Zip | BTH positive control plasmid; Amp^r^ | (6) |
| p25Zip | BTH positive control plasmid; Kan^r^ | (6) |
| p18PBP2 | pUT18C containing *cyaA*T18-*pbp2* fusion; Amp^r^ | (7) |
| p25PBP2 | pKT25 containing *cyaA*T25-*pbp2* fusion; Kan^r^ | (7) |
| pUT18-GpsB | pUT18 containing *gpsB*-*cya*AT18 fusion, Amp^R^ | This work |
| pKNT25-GpsB | pKNT25 with *gpsB*-*cya*AT25 fusion, Kan^R^ | This work |
| pKT25-GpsB | pKT25 containing *cya*AT25-*gpsB* fusion; Kan^R^ | This work |

Abbreviations: Amp^R^ – ampicillin resistance; Ery^R^ – erythromycin resistance Kan^R^ – Kanamycin resistance; cad – cadmium

Table 2: Bacterial strains used in this work

| Strain | Description | Reference |
| --- | --- | --- |
| *E. coli* | | |
| DC10B | *dam+ dcm+ ∆hsdRMS endA1 recA1* | (8) |
| BTH101 | Reporter strain for BTH system; *cya^-^* | (6) |
| *S. aureus* | | |
| RN4220 | Restriction deficient derivative of NCTC8325-4 | (9) |
| JE2 | CA-MRSA | (10) |
| COL | HA-MRSA | (11) |
| JE2 Δ*1090* | JE2 SAUSA300_1090 deletion mutant | This work |
| JE2 Δ*ssaA* | JE2 *ssaA* deletion mutant | This work |
| JE2 Δ*pbpC* | JE2 *pbpC* (encoding PBP3) deletion mutant | (2) |
| JE2 Δ*rodA* | JE2 *rodA* deletion mutant | This work |
| JE2 Δ*0128* | JE2 SAUSA300_0128 deletion mutant | This work |
| JE2 Δ*mreD* | JE2 *mreD* deletion mutant | This work |
| JE2 Δ*gpsB* | JE2 *gpsB* deletion mutant | This work |
| JE2 Δ*pknB* | JE2 *pknB* deletion mutant | This work |
| JE2 Δ*mreC* | JE2 *mreC* deletion mutant | This work |
| JE2 Δ*rodZ* | JE2 *rodZ* deletion mutant | This work |
| JE2 Δ*pbp4* | JE2 *pbpD* deletion mutant | This work |
| JE2 pCNX | JE2 transformed with pCNX empty vector; Kan^R^ | This work |
| JE2 Δ*1090* pCNX*1090* | JE2 SAUSA300_1090 deletion mutant transformed with pCNX*1090*; Kan^R^ | This work |
| JE2 Δ*ssaA* pCNX*ssaA* | JE2 *ssaA* deletion mutant transformed with pCNX*ssaA*; Kan^R^ | This work |
| JE2 Δ*0128* pCNX*0128* | JE2 SAUSA300_0128 deletion mutant transformed with pCNX*0128*; Kan^R^ | This work |
| JE2 Δ*mreD* pCNX*mreD* | JE2 *mreD* deletion mutant transformed with pCNX*mreD*; Kan^R^ | This work |
| JE2 Δ*gpsB* pCNX*gpsB* | JE2 *gpsB* deletion mutant transformed with pCNX*gpsB*; Kan^R^ | This work |
| JE2 Δ*pknB* pCNX*pknB* | JE2 *pknB* deletion mutant transformed with pCNX*pknB*; Kan^R^ | This work |
| JE2 Δ*mreC* pCNX*mreC* | JE2 *mreC* deletion mutant transformed with pCNX*mreC*; Kan^R^ | This work |
| JE2 Δ*rodZ* pCNX*rodZ* | JE2 *rodZ* deletion mutant transformed with pCNX*rodZ*; Kan^R^ | This work |
| JE2 Δ*pbp4* pCNX*pbp4* | JE2 *pbpD* deletion mutant transformed with pBCBPM115; Kan^R^ | This work |
| JE2 *lgt*::ΦΝΣ | JE2 with a transposon inserted in the *lgt* gene (Nebraska transposon mutant library); Ery^R^ | (10) |
| COL Δ*gpsB* | COL *gpsB* deletion mutant | This work |
| COL Δ*gpsB* pCNX*gpsB* | COL *gpsB* deletion mutant transformed with pCNX*gpsB*; Kan^R^ | This work |
| COL Δ*pbp4* | COL *pbpD* deletion mutant | This study |
| COL Δ*pbp4ΔgpsB* | COL *pbpD* and *gpsB* deletion mutant | This study |
| ColΔ*rodAΔpbpC* | COL *rodA* and *pbpC* (encoding PBP3) deletion mutant | (2) |
| ColsGFP-PBP1 | COL *pbpA::sgfp-pbpA* | (12) |
| BCBPM073 | COL *pbpB::sgfp-pbpB* | (13) |
| ColsGFP-PBP3 | COL *pbpC::sgfp-pbpC* | (12) |
| COLpPBP4-YFP | COL *pbpD::ppbpDyfp;* Kan^R^ (*ppbpDyfp* is an pMutinYFP derivative) | (14, 15) |
| COL EzrA-sGFP | COL *ezrA::ezrA-sgfp* | (16) |
| ColsGFP-PBP1 Δ*gpsB* | COL *pbpA::sgfp-pbpA ΔgpsB* | This work |
| ColsGFP-PBP2 Δ*gpsB* | COL *pbpB::sgfp-pbpB ΔgpsB* obtained by deleting *gpsB* in BCBPM073 | This work |
| ColsGFP-PBP3 Δ*gpsB* | COL *pbpC::sgfp-pbpC ΔgpsB* | This work |
| COLpPBP4-YFP Δ*gpsB* | COL *pbpD::ppbpDyfp ΔgpsB* Kan^R^ | This work |
| COL EzrA-sGFP Δ*gpsB* | COL *ezrA::ezrA-sgfp* *ΔgpsB* | This work |
| ColsGFP-PBP2 pCNX*gpsB* | COL *pbpB::sgfp-pbpB* transformed with pCNX*gpsB;* Kan^R^ | This work |
| ColsGFP-PBP2 Δ*gpsB* pCNX*gpsB* | COL *pbpB::sgfp-pbpB ΔgpsB* transformed with pCNX*gpsB;* Kan^R^ | This work |
| COLpPBP4-YFP pCN51*gpsB* | COL *pbpD::ppbpDyfp* transformed with pCN51*gpsB;* Ery^R^ | This work |
| COLpPBP4-YFP Δ*gpsB* pCN51*gpsB* | COL *pbpD::pbpD-yfp ΔgpsB* transformed with pCN51*gpsB;* Ery^R^ | This work |

Abbreviations: Amp^R^ – ampicillin resistance; Ery^R^ – erythromycin resistance Kan^R^ – Kanamycin resistance

Table 3: Primers used in this work

| Primer | Used for | Insert length (bp) | Sequence 5’-3’ |
| --- | --- | --- | --- |
| pMAD_2_Up_ 1090_fwd | Amplification of upstream and downstream regions of 1090 gene to clone into pMAD plasmid | 996 | CGATGCATGCCATGGTACCCCAATTAAGTGTAGACGATTC |
| Up_down_1090_rev |  |  | GCACGACACAATTACTTAACCTCCTTCTCC |
| Up_down_1090_fwd |  | 1000 | GGTTAAGTAATTGTGTCGTGCTATAATTACG |
| down1090_ pMAD_rev |  |  | CTTCTAGAATTCGAGCTCCCCAAGCATATGAAAACTTATTTATCATTC |
| pMAD_up_ ssaA_fwd | Amplification of upstream and downstream regions of *ssaA* gene to clone into pMAD plasmid | 1000 | CGATGCATGCCATGGTACCCCAACACACATGTAATTAATAATCTTATC |
| up_down_ssaA_rev |  |  | CATAGCCATCACTAATTTAAAAATATCCTCCTAAAAATTTTAAATC |
| up_down_ssaA_fwd |  | 1000 | GGAGGATATTTTTAAATTAGTGATGGCTATGTTTACGC |
| down_ssaA_ pMAD |  |  | CTTCTAGAATTCGAGCTCCCGACAGATGGTTTCAAAGC |
| pMAD_up_0128_fwd2 | Amplification of upstream and downstream regions of *0128* gene to clone into pMAD plasmid | 937 | GATGCATGCCATGGTACCCCGCAAGTTATAGTGGTTGTTG |
| up_down_0128_rev2 |  |  | CTCGATTAGTTTGTCACCTTAAATATTCTCTTTATTCTTTAGTG |
| up_down_0128_fwd |  | 1000 | GGGAATAGACTGACAAACTAATCGAGAGAC |
| down_0128_ pMAD_rev |  |  | CTTCTAGAATTCGAGCTCCCAGTGGATGATTATTAACGG |
| upGpsB P1N | Amplification of upstream and downstream regions of *gpsB* gene to clone into pMAD plasmid | 621 | cttaccatggggcgtaacaaaagagggtac |
| downGpsB P3 |  |  | cttttgtatttagtaattacattttttccacctcattagaaac |
| upGpsB P2 |  | 973 | gtttctaatgaggtggaaaaaatgtaattactaaatacaaaag |
| downGpsB P2 B |  |  | cgaggatccgatttaacgcttctaccttg |
| pMAD_dPknB_Up_Fwd | Amplification of upstream and downstream regions of *pknB* gene to clone into pMAD plasmid | 805 | CTATCGATGCATGCCATGGTACCCGTAAAGACAAATGCTAGAGG |
| dPknB_Up_Rev |  |  | TTATACATCATCATATATTTTACCTATCATACTTTATCACCTTCAATAGCCGCGA |
| dPknB_Down_Fwd |  | 773 | ATGATAGGTAAAATATATGATGATGTATAAATATAATTGAAGTAAATGTACCGA |
| pMAD_dPknB_Down_Rev |  |  | GAAGCTTCTAGAATTCGAGCTCCCCCACTAAGTACTATAAGTCCAG |
| RodZ_P1_EcoRI | Amplification of upstream and downstream regions of *rodZ* gene to clone into pMAD plasmid | 850 | gctGAATTCcaccatgacagttgcagag |
| RodZ_P2 |  |  | atttctgttattcatagcctccttacacttac |
| RodZ_P3 |  | 841 | ggaggctatgaataacagaaataaattagtgag |
| RodZ_P4_NcoI |  |  | tgcattCCATGGttatatcccccttgtatcg |
| PBP4_KO-P1 | Amplification of upstream and downstream regions of *pbp4* gene to clone into pMAD plasmid | 1004 | tgctccatggagattgtgtacttgtcg |
| PBP4_KO-P2 |  |  | tccgtttttagtatgttaaagcgttaatcttccct |
| PBP4_KO-P3 |  | 1018 | agggaagattaacgctttaacatactaaaaacg |
| PBP4_KO-P4 |  |  | acgctggatccacaagtaacgatgaag |
| pMADI | Verification of insert in pMAD plasmid for sequencing | variable | CTCCTCCGTAACAAATTGAGG |
| pMADII |  |  | CGTCATCTACCTGCCTGGAC |
| pCNseqUPfwd | Verification of insert under P_cad_ promoter for sequencing | variable | CATATCAGGCAGATAATG |
| pCNseqDWRev |  |  | CAAAATTATACATGTCAACG |
| pCNX_rbs_ 1090_fwd | Amplification of *1090* gene to clone in pCNX plasmid | 981 | GTCGACTCTAGAggatccCCaatatctaaggaggtaatataATGGAGACTTATGAATTTAAC |
| 1090_pCNX_ rev |  |  | CCTGAATTCGAGCTCGGTACCCTTATGCATCTCTTTTTCGAATTG |
| pCNX_rbs_ ssaA_fwd | Amplification of *ssaA* gene to clone in pCNX plasmid | 866 | GTCGACTCTAGAggatccCCaatatctaaggaggtaatataATGAAGAAAATCGCTACAGC |
| ssaA_pCNX_ rev |  |  | CTGAATTCGAGCTCGGTACCCTTAGTGAATGAAGTTATAACCAG |
| pCNX_rbs_ 0128_fwd | Amplification of *0128* gene to clone in pCNX plasmid | 687 | GTCGACTCTAGAggatccCCatatctaaggaggtaatatATGGAAAAAAATGTAGAAAAATCATTC |
| 0128_pCNX_ rev |  |  | CTGAATTCGAGCTCGGTACCCTCAATCTTTTTTCGAGACATGG |
| pCNX_rbs_ gpsB_fwd | Amplification of *gpsB* gene to clone in pCNX plasmid | 408 | GTCGACTCTAGAggatccCCaatatctaaggaggtaatataATGTCAGATGTTTCATTG |
| gpsB_pCNX_ rev |  |  | CTGAATTCGAGCTCGGTACCCTTATTTACCAAATACAGCTTTTTCTAAG |
| pCNX_PknB_Up_Fwd | Amplification of *pknB* gene to clone in pCNX plasmid | 2055 | GTCGACTCTAGAGGATCCCCGGCTATTGAAGGTGATAAAGTATGATAGG |
| pCNX_PknB_Down_Rev |  |  | AATTCGAGCTCGGTACCCTTATACATCATCATAGCTGAC |
| pMreC_SmaI_P1 | Amplification of *mreC* gene to clone in pCNX plasmid | 881 | tcccccggggacataatagaggtgttctg |
| BTH_mreC_EcoRI_P2 |  |  | CGGAATTCttatttatccctgctttc |
| pMreD_SmaI_P1 | Amplification of *rmreD* gene to clone in pCNX plasmid | 568 | \| tcccccggggatgaaagcagggataaataatg \| \| --- \| |
| BTH_mreD_Nfus_EcoRI_P2 |  |  | CGGAATTCttaccattgacgacgtttc |
| pCNX_rbs_ rodZ_fwd | Amplification of *rodZ* gene to clone in pCNX plasmid | 455 | GTCGACTCTAGAggatccCCaatatctaaggaggtaatatattgaaaacggtcggtgaag |
| rodZ_pCNX_ rev |  |  | CTGAATTCGAGCTCGGTACCCttaaaatattaaaactaacatgatccataac |
| up_1090_seq_ fwd | Verification of *1090* gene deletion | 2166 | CGAACTGACATTCGAGTG |
| down_1090_ seq_rev |  |  | GACAAGTTGCTACAAGTC |
| up_ssaA_seq_ fwd | Verification of *ssaA* gene deletion | 2110 | GACGACTACCATCGTATG |
| down_ssaA_ seq_rev |  |  | GTTGCGATAGTAGCTGTAG |
| up_0128_Seq Exc_fwd | Verification of *0128* gene deletion | 830 | CTGGAGGATCTGATTACG |
| down_0128_ Seq Exc_rev |  |  | CGATTGGTGTTGTTGGTC |
| up_mreD_Seq Exc_fwd | Verification of *mreD* gene deletion | 451 | CAAGTGGATTAGCTGATC |
| down_mreD_ SeqExc_rev |  |  | GTATCTCCTTCGTTTACGTC |
| up_gpsB_Seq Exc_fwd | Verification of *gpsB* gene deletion | 547 | CTAGTCACACTGGTACTC |
| up_gpsB_Seq Exc_fwd |  |  | CGAGTCCTAAGTTCTTCAAG |
| up_mreC_Seq Exc_fwd | Verification of *mreC* gene deletion | 440 | CACGTTGGAGACAACTTC |
| down_mreC_ SeqExc_rev |  |  | GCTGAGCAATAATGATACG |
| dPknB_Conf_Fwd | Verification of *pknB* gene deletion | 1914 | GAATGACCAACTAGAACATGC |
| dPknB_Conf_Rev |  |  | ACTGAATCCAGGTGTGTCT |
| up_rodZ_SeqExc_fwd | Verification of *rodZ* gene deletion | 1864 | ctagaagtgttactggaac |
| down_rodZ_SeqExc_rev |  |  | gcaataatggcaattgac |
| PBP4KO_P5 | Verification of *pbpD* gene deletion | 412 | \| acctttagctacacacg \| \| --- \| |
| PBP4KO_P6 |  |  | acatgactgggaaggtg |
| GpsB P3 PstI | Amplification of *gpsB* to clone in pUT18 and pKNT25 plasmids | 363 | gccCTGCAGgatgtcagatgtttcattg |
| GpsB P2 KpnI |  |  | gccGGTACCgctttaccaaatacagctttttc |
| GpsB_P1 PstI | Amplification of *gpsB* to clone in pKT25 plasmid | 364 | cctCTGCAGccatgtcagatgtttcattg |
| GpsB_P2 KpnI |  |  | gccGGTACCgctttaccaaatacagctttttc |
| pUT18 P10 | pUT18 sequencing primer |  | CGATTTTCCACAACAAGTCG |
| pUT18 P11 | pUT18 sequencing primer |  | gttgtgtggaattgtgagcg |
| pKNT25 P3 | pKNT25 sequencing primer |  | cttccggctcgtatgttgtg |
| pKNT25 P14 | pKNT25 sequencing primer |  | CCTTGATGCCATCGAGTACG |
| pKT25 P1 | pKT25 sequencing primer |  | GTTCGCCATTATGCCGCATC |
| pKT25 P15 | pKT25 sequencing primer |  | gctgcaaggcgattaagttg |

Table 4: Selected mutants from Nebraska Transposon Mutant Library screening ranked by increasing eccentricity

| Screening Rank | Name | Gene Description | Acession # | Area (µm2) | Eccentricity | Phase (%) | | | Eccentricity of deletion mutants (Phase 3 cells only) | | | | | |
| --- | --- | --- | --- | --- | --- | --- | --- | --- | --- | --- | --- | --- | --- | --- |
|  |  |  |  |  |  |  |  |  | Replicate 1 | | Replicate 2 | | Replicate 3 | |
|  |  |  |  |  |  | 1 | 2 | 3 | Mean | Std | Mean | Std | Mean | Std |
| - | JE2 | - | - | 1.36 | 0.50 | 41 | 25 | 34 | 0.55 | 0.13 | 0.53 | 0.13 | 0.56 | 0.10 |
| 1 | SAUSA300_ 1090 | hypothetical protein | SAUSA300_1090 | 1.17 | 0.44 | 53 | 25 | 20 | 0.51 | 0.12 | 0.54 | 0.12 | 0.54 | 0.14 |
| 2* | *lgt* | prolipoprotein diacylglyceryl transferase | SAUSA300_0744 | 1.23 | 0.44 | 47 | 24 | 27 | 0.60 | 0.12 | 0.61 | 0.12 | 0.59 | 0.13 |
| 3 | *ssaA* | secretor antigen precursor SsaA | SAUSA300_2249 | 1.25 | 0.44 | 51 | 24 | 24 | 0.53 | 0.14 | 0.51 | 0.14 | 0.49 | 0.14 |
| 4 | *pbp3* (*pbpC*) | penicillin-binding protein 3 | SAUSA300_1512 | 1.28 | 0.45 | 50 | 21 | 27 | Published (2) | | | | | |
| 5 | *rodA* | rod shape-determining protein RodA | SAUSA300_2040 | 1.29 | 0.45 | 52 | 16 | 30 |  |  |  |  |  |  |
| 6 | SAUSA300_0128 | hypothetical protein | SAUSA300_0128 | 1.13 | 0.45 | 52 | 27 | 19 | 0.53 | 0.13 | 0.51 | 0.12 | 0.52 | 0.12 |
| 7 | *mreD* | rod shape-determining protein MreD | SAUSA300_1604 | 1.21 | 0.45 | 41 | 22 | 35 | 0.53 | 0.13 | 0.51 | 0.12 | 0.55 | 0.12 |
| 67 | *gpsB* | cell cycle protein | SAUSA300_1337 | 1.27 | 0.45 | 41 | 23 | 34 | 0.46 | 0.12 | 0.47 | 0.12 | 0.46 | 0.12 |
| 74 | *pknB* | protein kinase | SAUSA300_1113 | 1.21 | 0.45 | 44 | 22 | 33 | 0.49 | 0.12 | 0.50 | 0.11 | 0.51 | 0.12 |
| 1150 | *mreC* | rod shape-determining protein MreC | SAUSA300_1605 | 1.16 | 0.48 | 47 | 27 | 25 | 0.55 | 0.13 | 0.51 | 0.13 | 0.52 | 0.13 |
| 1532 | *pbp4* (*pbpD*) | penicillin-binding protein 4 | SAUSA300_0629 | 1.36 | 0.49 | 48 | 20 | 30 | 0.51 | 0.10 | 0.53 | 0.11 | 0.53 | 0.11 |
| N.A. | *rodZ* | hypothetical protein | SAUSA300_1175 | Not part of Nebraska Library | | | | | 0.50 | 0.11 | 0.50 | 0.11 | 0.50 | 0.11 |

**lgt* transposon mutant was a false positive

**Supplementary Figure 1: Violin plot of the overall distribution of eccentricity values for the entire NTML library**. Eccentricity values for the 11 mutants selected for this study (described in Table 4) are shown in blue.


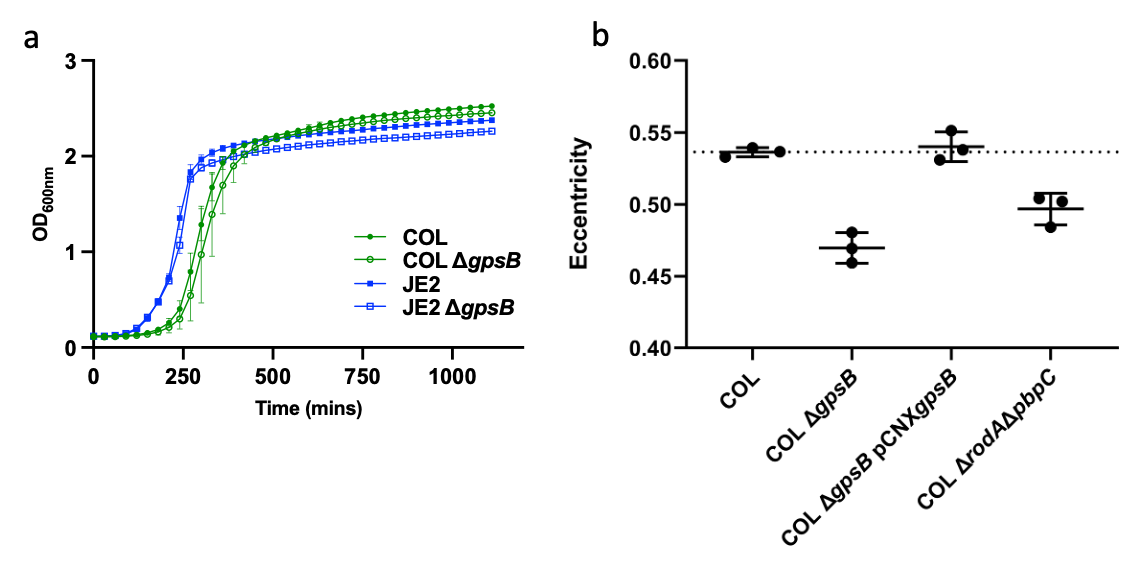


Supplementary Figure 2: GpsB is not essential for viability, but is required for elongation of *S. aureus* strain COL cells. a. Growth of COL and JE2 parental strains, and corresponding *gpsB* deletion mutants was assessed at 37ºC, showing that lack of GpsB does not impair growth. b. Cell eccentricity was measured in cells with a complete septum (cell cycle phase 3) of strains COL, COL Δ*gpsB* and complemented strain COL Δ*gpsB* pCNX *gpsB.* Strain COLΔ*rodA* Δ*pbpC*, lacking RodA and PBP3, previously reported to have more spherical cells (2) was used for comparison. n>400 cells/replicate, 3 replicates per strain. Black lines represent the mean eccentricity and standard deviation for each strain. The average eccentricity of COL cells is indicated by the dashed line.


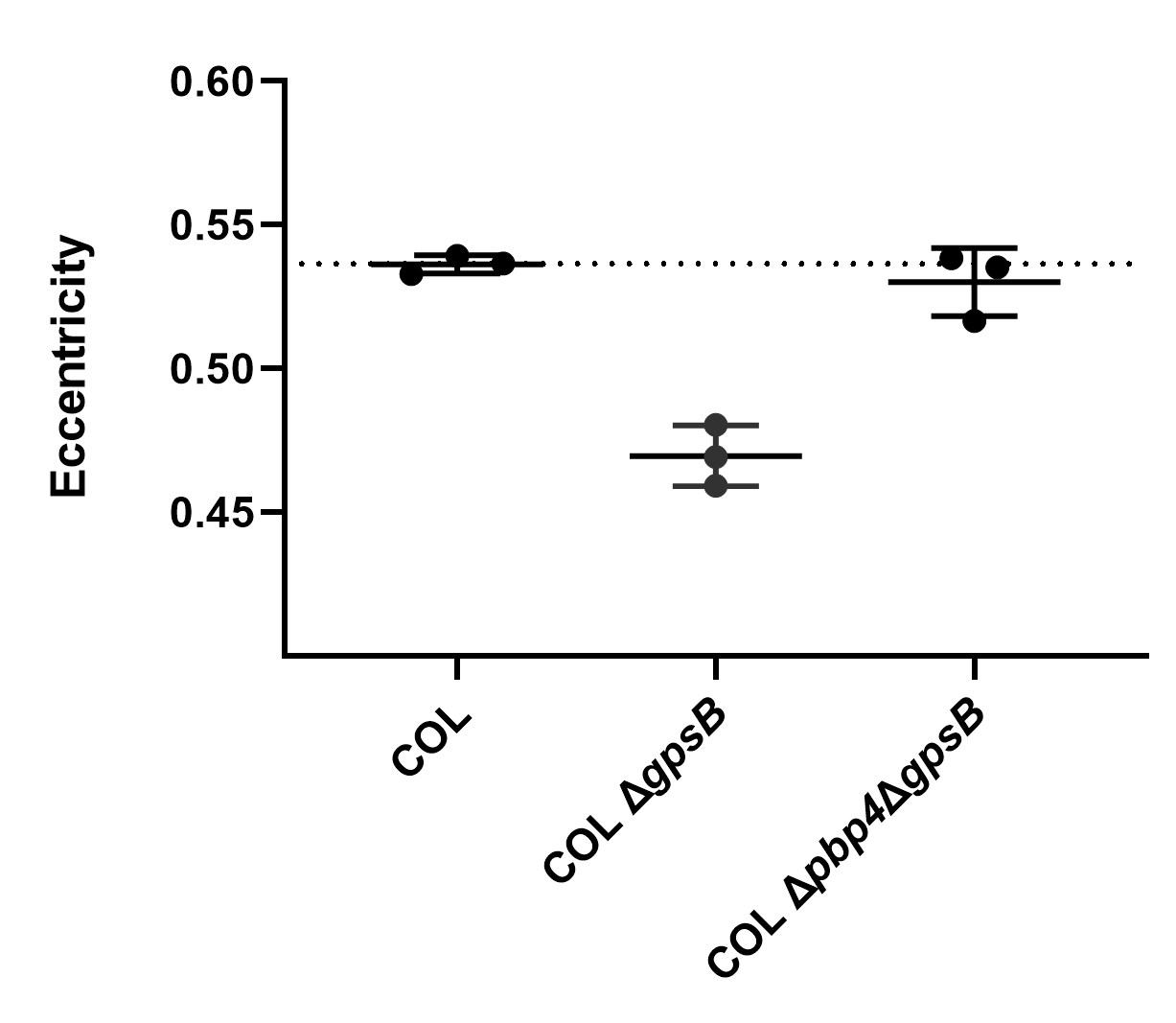


**Supplementary Figure 3: PBP4 peripheral peptidoglycan crosslinking activity is required for decreased eccentricity of cells lacking GpsB.** Cell eccentricity was measured in cells with a complete septum (cell cycle phase 3) of strains COL (n>550/replicate), COL Δ*gpsB* (n>500/replicate) and COL Δ*gpsB*Δ*pbp4* (n>150/replicate)*.* Three replicates were made per strain. Black lines represent the mean eccentricity and standard deviation for each strain. The average eccentricity of COL cells is indicated by the dashed line.

BIBLIOGRAPHY

1. Arnaud M, Chastanet A, Debarbouille M. 2004. New vector for efficient allelic replacement in naturally nontransformable, low-GC-content, gram-positive bacteria. Appl Environ Microbiol 70:6887-6891.

2. Reichmann NT, Tavares AC, Saraiva BM, Jousselin A, Reed P, Pereira AR, Monteiro JM, Sobral RG, VanNieuwenhze MS, Fernandes F, Pinho MG. 2019. SEDS-bPBP pairs direct lateral and septal peptidoglycan synthesis in *Staphylococcus aureus*. Nat Microbiol 4:1368-1377.

3. Tavares AC, Fernandes PB, Carballido-Lopez R, Pinho MG. 2015. MreC and MreD proteins are not required for growth of *Staphylococcus aureus*. PLoS One 10:e0140523.

4. Monteiro JM, Fernandes PB, Vaz F, Pereira AR, Tavares AC, Ferreira MT, Pereira PM, Veiga H, Kuru E, VanNieuwenhze MS, Brun YV, Filipe SR, Pinho MG. 2015. Cell shape dynamics during the staphylococcal cell cycle. Nat Commun 6:8055.

5. Charpentier E, Anton AI, Barry P, Alfonso B, Fang Y, Novick RP. 2004. Novel cassette-based shuttle vector system for gram-positive bacteria. Appl Environ Microbiol 70:6076-6085.

6. Karimova G, Pidoux J, Ullmann A, Ladant D. 1998. A bacterial two-hybrid system based on a reconstituted signal transduction pathway. Proc Natl Acad Sci U S A 95:5752-5756.

7. Reed P, Veiga H, Jorge AM, Terrak M, Pinho MG. 2011. Monofunctional transglycosylases are not essential for *Staphylococcus aureus* cell wall synthesis. Journal of Bacteriology 193:2549-2556.

8. Monk IR, Shah IM, Xu M, Tan MW, Foster TJ. 2012. Transforming the untransformable: application of direct transformation to manipulate genetically *Staphylococcus aureus* and *Staphylococcus epidermidis*. MBio 3:e00277-11.

9. Nair D, Memmi G, Hernandez D, Bard J, Beaume M, Gill S, Francois P, Cheung AL. 2011. Whole-genome sequencing of *Staphylococcus aureus* strain RN4220, a key laboratory strain used in virulence research, identifies mutations that affect not only virulence factors but also the fitness of the strain. J Bacteriol 193:2332-2335.

10. Fey PD, Endres JL, Yajjala VK, Widhelm TJ, Boissy RJ, Bose JL, Bayles KW. 2013. A genetic resource for rapid and comprehensive phenotype screening of nonessential *Staphylococcus aureus* genes. MBio 4:e00537-12.

11. Gill SR, Fouts DE, Archer GL, Mongodin EF, Deboy RT, Ravel J, Paulsen IT, Kolonay JF, Brinkac L, Beanan M, Dodson RJ, Daugherty SC, Madupu R, Angiuoli SV, Durkin AS, Haft DH, Vamathevan J, Khouri H, Utterback T, Lee C, Dimitrov G, Jiang L, Qin H, Weidman J, Tran K, Kang K, Hance IR, Nelson KE, Fraser CM. 2005. Insights on evolution of virulence and resistance from the complete genome analysis of an early methicillin-resistant *Staphylococcus aureus* strain and a biofilm-producing methicillin-resistant *Staphylococcus epidermidis* strain. J Bacteriol 187:2426-2438.

12. Monteiro JM, Pereira AR, Reichmann NT, Saraiva BM, Fernandes PB, Veiga H, Tavares AC, Santos M, Ferreira MT, Macario V, VanNieuwenhze MS, Filipe SR, Pinho MG. 2018. Peptidoglycan synthesis drives an FtsZ-treadmilling-independent step of cytokinesis. Nature 554:528-532.

13. Tan CM, Therien AG, Lu J, Lee SH, Caron A, Gill CJ, Lebeau-Jacob C, Benton-Perdomo L, Monteiro JM, Pereira PM, Elsen NL, Wu J, Deschamps K, Petcu M, Wong S, Daigneault E, Kramer S, Liang L, Maxwell E, Claveau D, Vaillancourt J, Skorey K, Tam J, Wang H, Meredith TC, Sillaots S, Wang-Jarantow L, Ramtohul Y, Langlois E, Landry F, Reid JC, Parthasarathy G, Sharma S, Baryshnikova A, Lumb KJ, Pinho MG, Soisson SM, Roemer T. 2012. Restoring methicillin-resistant *Staphylococcus aureus* susceptibility to β-lactam antibiotics. Sci Transl Med 4:126ra35.

14. Loskill P, Pereira PM, Jung P, Bischoff M, Herrmann M, Pinho MG, Jacobs K. 2014. Reduction of the peptidoglycan crosslinking causes a decrease in stiffness of the *Staphylococcus aureus* cell envelope. Biophys J 107:1082-1089.

15. Atilano ML, Pereira PM, Yates J, Reed P, Veiga H, Pinho MG, Filipe SR. 2010. Teichoic acids are temporal and spatial regulators of peptidoglycan cross-linking in *Staphylococcus aureus*. Proc Natl Acad Sci U S A 107:18991-18996.

16. Saraiva BM, Sorg M, Pereira AR, Ferreira MJ, Caulat LC, Reichmann NT, Pinho MG. 2020. Reassessment of the distinctive geometry of *Staphylococcus aureus* cell division. Nat Commun 11:4097.
